# The Extraction of Depth Structure from Shading and Texture in the Macaque Brain

**DOI:** 10.1371/journal.pone.0008306

**Published:** 2009-12-14

**Authors:** Koen Nelissen, Olivier Joly, Jean-Baptiste Durand, James T. Todd, Wim Vanduffel, Guy A. Orban

**Affiliations:** 1 Laboratorium voor Neuro- & Psychofysiologie, K. U. Leuven Medical School, Leuven, Belgium; 2 Department of Psychology, Ohio State University, Columbus, Ohio, United States of America; 3 Athinoula A. Martinos Center for Biomedical Imaging, Massachusetts General Hospital, Charlestown, Massachusetts, United States of America; 4 Department of Radiology, Harvard Medical School, Boston, Massachusetts, United States of America; Rutgers University, United States of America

## Abstract

We used contrast-agent enhanced functional magnetic resonance imaging (fMRI) in the alert monkey to map the cortical regions involved in the extraction of 3D shape from the monocular static cues, texture and shading. As in the parallel human imaging study [1], we contrasted the 3D condition to several 2D control conditions. The extraction of 3D shape from texture (3D SfT) involves both ventral and parietal regions, in addition to early visual areas. Strongest activation was observed in CIP, with decreasing strength towards the anterior part of the intraparietal sulcus (IPS). In the ventral stream 3D SfT sensitivity was observed in a ventral portion of TEO. The extraction of 3D shape from shading (3D SfS) involved predominantly ventral regions, such as V4 and a dorsal potion of TEO. These results are similar to those obtained earlier in human subjects and indicate that the extraction of 3D shape from texture is performed in both ventral and dorsal regions for both species, as are the motion and disparity cues, whereas shading is mainly processed in the ventral stream.

## Introduction

The processing of 3D shape is important for human and nonhuman primates as they live in a 3D world. The third dimension of shape or depth structure can be recovered from several cues [2]. These include differences among multiple images that can arise from binocular disparity and motion, and also static monocular cues, such as texture and shading. Relative to the other possible sources of information, shading has received little attention in studies of the monkey visual system, although it has been shown behaviorally that macaques can use this cue to recover depth structure [3]. Single cell studies have shown that V4 neurons can represent the direction of illumination, a parameter important for the interpretation of shading patterns. Yamane et al. [4] have shown that TE neurons can signal the 3D shape of surfaces defined by disparity and monocular static cues. While this study showed that individual TE neurons can use the shading cue to signal 3D shape, it is unclear to what extend shading contributes to the selectivity of TE neurons for 3D shape described in that report since shading was systematically combined with other cues, disparity and texture. In a recent human functional imaging study, Georgieva et al. [1] showed that only a single region of the human brain in the ventral pathway, the caudal ITG part of the LO complex, was activated selectively by the presentation of 3D shapes defined by shading as compared to a range of 2D control stimuli. Thus a first aim of the present study was to identify the equivalent region in macaque monkeys, so as to possibly target it subsequently with micro-electrode recordings and to provide further evidence for homologous areas in the two species.

The second monocular static cue for depth structure, texture, has been investigated predominantly by using first order gradients of texture. Neurons in CIP [5], [6], in TEs [7] and to some extent in MT/V5 [8] are selective for the orientation of texture gradients. There is also some evidence from a fMRI study in anaesthetized macaques [9] that both dorsal and ventral visual regions contribute to the extraction of depth structure from texture in monkeys. Intriguingly this latter study failed to report activation of CIP in the contrast 3D shape from texture compared to scrambled controls. Human imaging has also revealed the implication of dorsal and ventral visual regions in the extraction of depth structure in curved 3D surfaces from texture [1]. However, this latter study reported only a single activation site in the ventral visual pathway, in the vicinity of the shading activation site. On the contrary the Sereno et al. study [9] reported multiple sites in the ventral pathway. Thus it is unclear whether regions involved in extraction of depth structure from texture are similar or not in the human and non human primates and whether these regions in the monkey match what is known from single cell studies. The second aim of our study was therefore to compare the extraction of depth structure from texture in alert macaque monkeys to what has been observed in humans with similar stimuli.

To pursue these two aims we used contrast enhanced fMRI in the awake macaque [10], [11] to map the visual cortical regions involved in the extraction of depth (or 3D) structure from shading (3D SfS) and from texture (3D SfT), using exactly the same stimuli as used in the human fMRI study of Georgieva et al. [1].

## Methods

### Subjects

Three (M1, M3, M5) male rhesus monkeys (4–6 kg, 3–5 years of age) participated in the experiments. All three monkeys participated in the 3D SfS experiment. Two of them (M1 and M5) participated in the 3D SfT experiment. All animal care and experimental procedures met the national and European guidelines and were approved by the ethical committee of the K.U.Leuven medical school. The details of the surgical procedures, training of monkeys, image acquisition, eye monitoring and statistical analysis of monkeys scans have been described previously [10], [12], [13], and will be described only briefly here. Monkeys sat in a sphinx position in a plastic monkey chair directly facing the screen. A plastic headpost was attached to the skull using C&B Metabond adhesive cement (Parkell, Inc, New York) together with Palacos R+G bone cement and ∼15 ceramic screws (Thomas recording). Throughout the training and testing sessions, the monkey's head was restrained by attaching the implanted headpost to the magnet compatible monkey chair (see [10] for details). Thus, during the tests, the monkeys were able to move all body parts except their head. It is important to note, however, that body movements are usually infrequent when the monkeys perform a task, be it a fixation task, during the scanning. A receiver only surface coil was positioned just above the head.

During training the monkeys were required to maintain fixation within a 2×2° window centered on a red dot (0.35×0.35°) in the center of the screen. Eye position was monitored at 60 Hz through pupil position and corneal reflection. During scanning the fixation window was slightly elongated in the vertical direction to 3°, to accommodate an occasional artifact on the vertical eye trace induced by the scanning sequence. The monkeys were rewarded (fruit juice) for maintaining their gaze within the fixation window for long periods (up to 6 s, eye blinks were ignored), while stimuli were projected in the background. With this strategy monkeys made 5–16 saccades per minute, each monkey exhibiting a relatively stable number of saccades over the different sessions/runs: 15/min for M1, 8/min for M3 and 16/min for M5 in the 3D SfS experiment and 10/min for M1 and 5/min for M3 in the 3D SfT experiment. Thus most monkeys were close to the human average in this regard and made about one saccade every 6 s. In no experiment was the number of saccades made by individual subjects significantly (p<0.05) different between the experimental conditions, except for the 3D SfT experiment in M1, where the number of saccades during fixation baseline (7/min) was lower than the three other experimental conditions (11/min). Between the three experimental conditions the number of saccades was not different in this experiment.

Before each scanning session, a contrast agent, monocrystalline iron oxide nanoparticle (MION), was injected into the monkey's femoral/saphenous vein (6–12 mg/kg). The use of the contrast agent improved both the contrast-noise ratio (by approximately fivefold) and the spatial selectivity of the magnetic resonance (MR) signal changes, compared to blood–oxygen-level dependent (BOLD) measurements [10], [11]. While BOLD measurements depend on blood volume, blood flow, and oxygen extraction, MION measurements depend only on blood volume [14]. For the sake of clarity, the polarity of the MION MR signal changes, which are negative for increased blood volumes, was inverted.

### Stimuli

**Figure 1:**
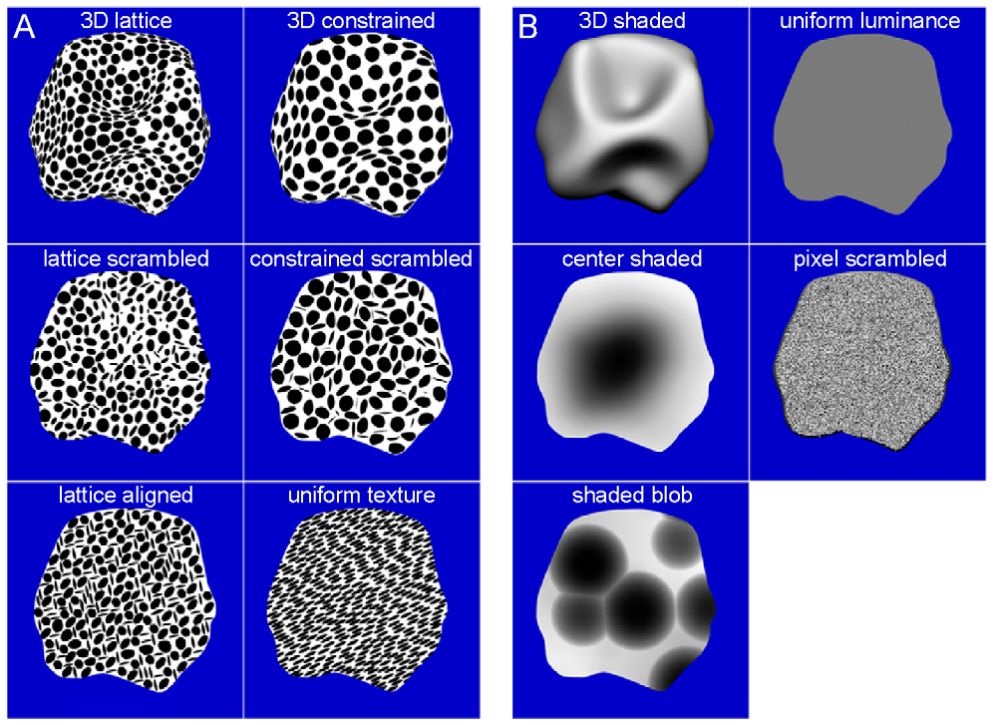
Visual stimuli. (A) Texture stimuli, from top to bottom, left column: 3D lattice, lattice scrambled, and lattice aligned; right column: 3D constrained, constrained scrambled and uniform texture. (B) Shading stimuli, from top to bottom, left column: 3D shaded, center shaded, shaded blob; right column: uniform luminance, and pixel scrambled.

Visual stimuli were projected from a liquid crystal display projector (Barco Reality 6400i, 1024×768, 60 Hz refresh frequency) onto a translucent screen positioned in the bore of the magnet at a distance of 56 cm from the point of observation. Subjects viewed the screen directly. The visual stimuli were exactly the same as those used by Georgieva et al. [1]. These stimuli were created and rendered using 3D Studio Max. They depicted 11 randomly generated complex 3D surfaces, representing the front surface of meaningless 3D objects, with a large assortment of variably shaped hills, ridges, valleys, and dimples, at multiple scales (see [15]–[17]). The images of these complex surfaces were presented on a blue background (34°×16.5°, 27.6 cd/m^2^). To quantitatively assess the variety of 3D structure in these displays Georgieva et al. [1] aligned all the surfaces in terms of size and position, and calculated a depth map for each image based on the 3D scene geometry that had been used to render it. They then correlated the depths at corresponding positions for each pairwise combination of surfaces. The resulting correlations produced r^2^ values that ranged from 0.02 to 0.44. The median of the distribution had a r^2^ of 0.184, and the first and third quartiles were 0.133 and 0.243, respectively. In other words, the different shapes we employed were largely independent of one another, with less than 20% overlap on average. This indicates that even if the overall 3D shape of the surfaces was convex, typical of most small objects, the variations around this average were large enough to create largely different 3D shapes. Additional variation was also created by presenting the displays at a variety of different sizes (5°–15°), as is shown in Figure S1. All of the surfaces were smoothly curved, so they did not provide information from configurations of edges and vertices (e.g. [18], [19]). Examples of the different stimulus types are presented in Figure 1A and B and a complete set of 3D shapes (with shading) is shown in Figure S1. When projected onto the translucent display screen in the bore of the magnet, the sizes of the depicted surfaces in the shading and texture stimuli averaged 10°.

In the 3D SfT experiment, the shapes were presented with 2 different types of volumetric texture that will be referred to, respectively, as the 3D lattice and 3D constrained conditions (Figure 1A). In both cases, the texture was composed of a set of small spheres that were distributed without overlapping in a 3D volume. Any region of the depicted surface that cut through a sphere was colored black, and any region that cut through the space between spheres was colored white. In the 3D lattice condition, the spheres were arranged in a hexagonal lattice within the texture volume. Note in this case that a local region of an object could cut through the center of a sphere, which would produce a large black dot on the object's surface, or it could just graze through the periphery of the sphere, which would produce a much smaller black dot. Thus, in the 3D lattice conditions, the depicted surfaces were covered with a pattern of circular polka dots that varied in size, and could be systematically aligned along the symmetry axes of the texture lattice. To eliminate these systematic alignments and variations of size, we also employed a 3D constrained condition, in which the spheres were distributed in 3D space such that their centers were constrained to lie on the depicted object surface at randomly selected positions. The impact of this constraint is that all of the polka dots on a depicted surface had the same size, and they were not systematically aligned with one another. We also included several control conditions in which the texture patterns did not produce a compelling perception of a 3D surface. These included transformed versions of the 3D lattice and constrained conditions, in which the positions of the texture elements were randomly scrambled within the boundaries of each object. These will be referred to, respectively, as the (2D) lattice-scrambled condition and the (2D) constrained-scrambled conditions (Figure 1A). The 3D lattice condition differs from its scrambled version not only by the presence of gradients but also by alignments of identical elements or patches of identical elements. In an effort to disentangle these properties, a (2D) lattice-aligned condition was included that eliminated the systematic texture gradients of the 3D lattice displays, but had a similar pattern of texture element alignments. Each stimulus contained 3–4 alignments of 3–6 identical elements. In the (2D) uniform texture condition all of the projected texture elements had the same circular or elliptic shape (Figure 1A). Although constant within a stimulus, the elements differed across stimuli: their size ranged from 0.12° to 1°, their elongation ranged from circular to 4/1 ratio and when elongated, elements also differed in orientation. Since the human imaging study [1] had shown that the two types of volumetric texture were redundant, we mainly used the lattice versions of the 3D and scrambled stimuli and the uniform texture conditions.

**Figure 2:**
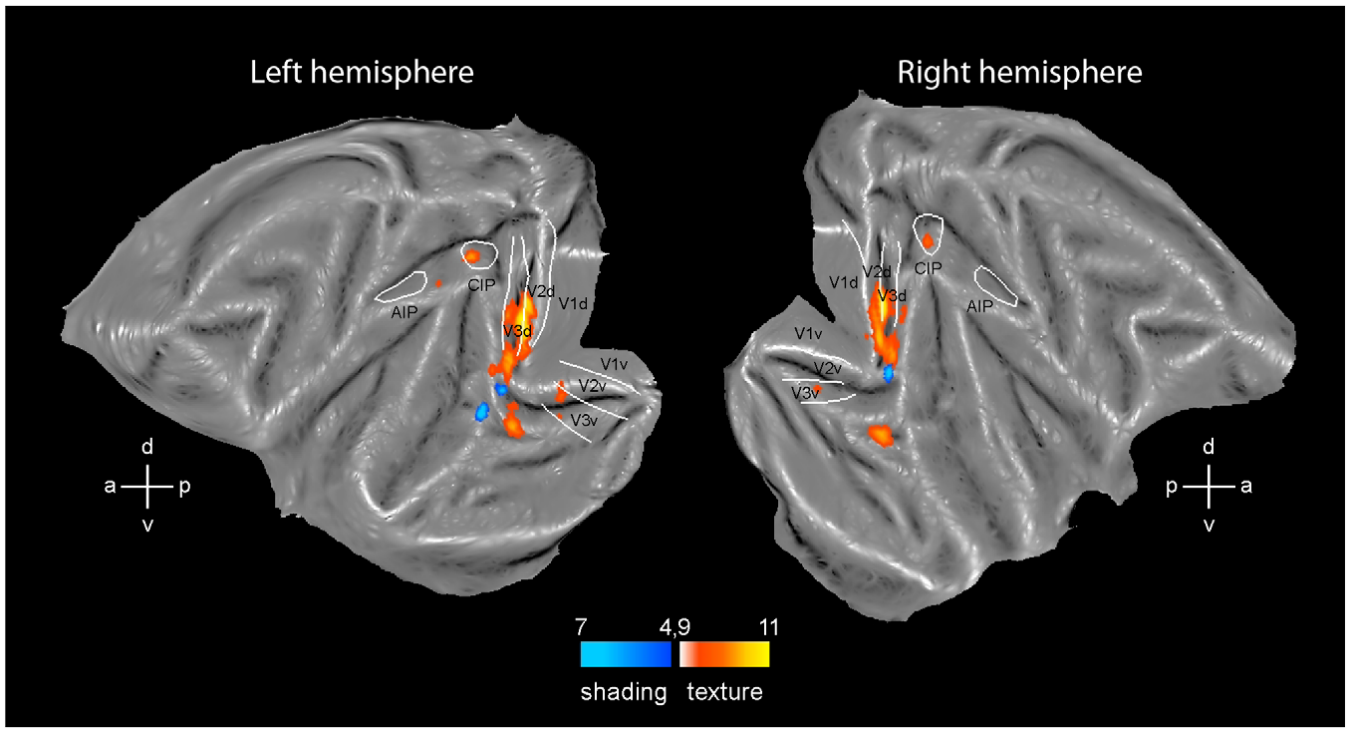
3D SfT and 3D SfS sensitive regions. Flatmaps of the left and right hemisphere of monkey template (M12) brain (Caret software) showing regions significant (fixed effects, p<0.05 corrected) in the conjunction of contrasts of the 3D SfT experiment (yellow to orange voxels, number of monkeys (n) = 2) and 3D SfS (blue voxels, n = 3) experiment. White lines indicate borders of V1-3 from Fize et al. [12] and of CIP and AIP from Durand et al. [22]. Color scales indicate t scores.

In the SfS experiment (Figure 1B and Figure S1), the surfaces in the 3D shaded condition (Figure S1) were illuminated by a rectangular area light at a 22° angle directly above the line of sight, and they were rendered using a standard Blinn reflectance model, in which the shading at each point is determined as a linear combination of its ambient, diffuse and specular components (mean luminance 367 cd/m^2^). In this experiment the reflectance was Lambertian, with no specular component. A number of control conditions were included in which the patterns of shading did not produce a compelling perception of a 3D surface, yet they had luminance histograms and/or Fourier amplitude spectra that were closely matched to those of the 3D displays (Fig. 2 from [1]). The first method we employed for eliminating the appearance of depth in the (2D) pixel scrambled condition was to randomly reposition the pixels (2.3×2.3 minarc) within the boundary of each object. The luminance histograms in these displays were identical to those in the 3D shaded condition, but the local luminance gradients were quite different. Note that the 3D shaded stimuli contained relatively large regions of nearly uniform luminance. The 2D uniform-luminance condition was designed to create flat looking stimuli that shared this aspect of the 3D displays. The stimuli in that condition included 11 silhouettes of different uniform luminance covering the same luminance range as in the 3D shaded condition (Figure 2A, vertical straight yellow bars in [1]). Two additional control conditions were created that attempted to mimic the pattern of shading gradients in the 3D displays without eliciting the appearance of a 3D surface. In the center-shaded condition, all stimuli had a luminance pattern that increased radially from the center of each silhouette. In the (2D) shaded-blob condition each silhouette contained 3–5 randomly shaped ovals with blurred edges on a light background. A 1-way analysis of variance (ANOVA) revealed that the luminance histograms in these latter 2 conditions did not differ significantly from that of the 3D condition. Finally, in this study we did not include the 2D unshaded-blob condition which in the human study [1] gave nearly identical results to the 2D shaded-blob condition.

It is important to keep in mind when evaluating the different control conditions for investigating the perception of 3D SfS or 3D SfT that it is not possible to create stimuli that are perceived as flat and share all the low-level 2D properties of the images used in the 3D conditions. The only viable solution to this problem is to use a wide battery of controls that collectively match the low-level properties of the 2D displays. This approach, which was also adopted in the present study, was validated by two quantitative analyses which were performed by Georgieva et al. [1] on the stimuli from the SfS experiment in order to compare their low-level properties. First, they compared the amplitude spectra of the 2D conditions with those of the 3D shaded condition. The amplitude spectra were calculated for each complete stimulus image, including the outline of the surface and the central part (15.4°×15.4°) of the background, using a 2D discrete fast Fourier transform (MATLAB). The 2D output (amplitude as a function of spatial frequency and orientation) was reduced to 1 dimension by collapsing across orientations. In general, the spectra of the 2D conditions differed slightly from that of the 3D condition, but the difference reached significance only for the pixel-scrambled and center-shaded conditions (Fig. 2B in [1]). Differences were most significant for the pixel-scrambled condition at high spatial frequencies, which is a typical feature of scrambling.

A second analysis examined the mean luminance distribution across the images in the different conditions (see Fig. 3 in [1]). The 3D shaded condition had a clear upper-lower asymmetry introduced by the position of the light source. This was much less the case for the 2 blob conditions. The center-shaded condition had luminance distribution that was much lower than that of the 3D shaded condition in the central part of the image. The luminance distributions of the last 2 conditions were also more symmetrical, as the center-shaded condition, but luminance levels were more similar to those of the 3D shaded conditions in the center of the images. These 2 analyses numerically show that it is impossible to create a single condition that is perceived as flat and shares all the low-level features of the images in the 3D condition. Even the shaded-blob condition does not meet all the requirements. This underscores the strength of the approach combining several control conditions, which was also followed in the present experiment. This of course implies that the statistical analysis requires all contrasts to be significant in the same voxel. That is the purpose of the conjuction analysis used in the present study.

In addition to the conditions described above, all of the experiments included a fixation-only condition to provide a baseline level of activation. All the experiments used block designs with block duration of 36 s, corresponding to 15 functional volumes or scans, and 12 blocks per time-series (or run). Within a block, the 11 stimuli were presented twice for 1400 ms, the remaining time being filled with 4 additional randomly selected stimuli (last one cut-off at 1 s). In the 3D SfT experiment the four conditions (3D lattice, lattice scrambled, uniform and fixation only) were repeated 2 times in a time-series, yielding 12 (3×4) blocks. In the 3D SfS experiment the six conditions (3D shaded, center shaded, shaded blob, uniform, pixel scrambled and fixation only) were repeated once, yielding also 12 (2×6) blocks.

### FMRI Data Acquisition and Analysis

The MRI images were acquired in a 1.5-T Sonata MR scanner (Siemens, Erlangen, Germany) using a surface coil. Each functional time series consisted of gradient-echo echoplanar whole-brain images (repetition time (TR) 2.4 s; echo time (TE) 27 ms; 32 sagital slices, 2×2×2 mm voxels). For each subject a T1-weighted anatomical (three-dimensional magnetization prepared rapid acquisition gradient echo, MPRAGE) volume (1×1×1 mm voxels) was acquired under anesthesia in a separate session.

Thirty-two and forty-two time-series were recorded in the SfT (16 per monkey) and SfS (14 per monkey) experiments respectively and presentation order of the conditions was randomized (6 different orders for SfS and 3 different orders for SfT) between these time series. In total, 960 volumes/condition were analyzed in both the 3D SfT and the 3D SfS experiments.

Data were analyzed using statistical parametric mapping (SPM5) and Match software. Only those runs were analyzed in which the monkeys maintained fixation within the window for >85% of the time and in which no significant differences in the numbers of saccades between conditions occurred. In these analyses, realignment parameters, as well as eye movement traces, were included as covariates of no interest to remove eye movement and brain motion artifacts. The realignment parameters provide quantitative information about the head motion in these experiments. In each monkey and each experiment the average (over the runs) x (medial-lateral), y (anterior-posterior) or z (dorsal-ventral) dimensions of head position differed by less than. 1 mm (x and y) or. 35 mm (z) from zero and the standard deviation was smaller than. 1 mm for x and y positions and less than 0.26 mm for the z position. The fMRI data of the monkeys were realigned and non-rigidly co-registered with the anatomical volumes of the template brain (M12, same as subject MM1 in [20]) using the Match software [21]. The algorithm computes a dense deformation field by composition of small displacements minimizing a local correlation criterion. Regularization of the deformation field was obtained by low-pass filtering. The functional volumes were then subsampled to 1 mm^3^ and smoothed with an isotropic Gaussian kernel [full width at half height, 1.5 mm]. Fixed effect group analyses were performed with an equal number of volumes per monkey, supplemented with single subject analysis. In order to include the different control conditions, we used a conjunction analysis, as in the human study [1]. The level of significance in the group analysis was set at p<0.05 family wise error (FWE) corrected for multiple comparisons. For descriptive purposes the level was lowered to that used in the human study (p<0.001 uncorrected). This level was also used in the single subject analyses. Activity profiles, plotting the % signal change with respect to fixation baseline, for the different experimental conditions, were calculated on small ROIs 7 voxels in size centered on the most significant voxel.

The fMRI data, registered onto the anatomy of M12, were mapped onto the macaque M12 flatmaps [22] using Caret software. Caret is available at http://www.nitrc.org/projects/caret/. The localization of visual regions (V1-3) was taken from the flatmaps in [12] and that of CIP and AIP was taken from the flattened IPS in [22]. The AIP ROI was anatomically defined and its posterior border with LIP coincided in [22] with the limit of the activation by saccades, the CIP ROI was defined from the 3D Structure-from-Depth activations with random lines (Fig. 1 in [22]).

## Results

### Cortical Regions Processing Depth Structure from Texture in the Monkey

We used the conjunction of two contrasts to map the regions processing 3D SfT: the contrast 3D lattice minus the 2D lattice scrambled condition and the contrast 3D lattice minus 2D uniform condition. The result for the group of two monkeys is shown in Figure 2 (yellow to orange voxels; p<0.05, corrected). Bilateral significant activation sites were observed in the early visual areas, in the posterior part of infero-temporal cortex and in the intraparietal sulcus (IPS). The early activation was located primarily in dorsal and ventral V3, but extended into V2 and V4. This activation spared the central representation and corresponds to the edge of the stimuli, as has been observed for the motion cue [23]. The ventral activation was located in the ventral portion of TEO centered on the PMTS. In the IPS the main activation site was located relatively posterior in the part corresponding to CIP of Durand et al. [22]. A second activation site was located more anteriorly in the lateral bank, just behind the AIP/LIP boundary [22], in the anterior part of LIP, in the left hemisphere. Lowering the threshold to p<0.001 (Figure S2) revealed a symmetrical activation in right hemisphere, as well as bilateral sites in posterior AIP and caudally in the medial bank. These latter sites might correspond to PIP as functionally defined in Durand et al. [22]. At p<0.001 uncorrected, unilateral activation sites were also observed in left MT/V5, left arcuate sulcus and left inferior frontal cortex. The arcuate site in the inferior ramus might correspond to F5a where depth structure from disparity has been reported [24].

**Figure 3:**
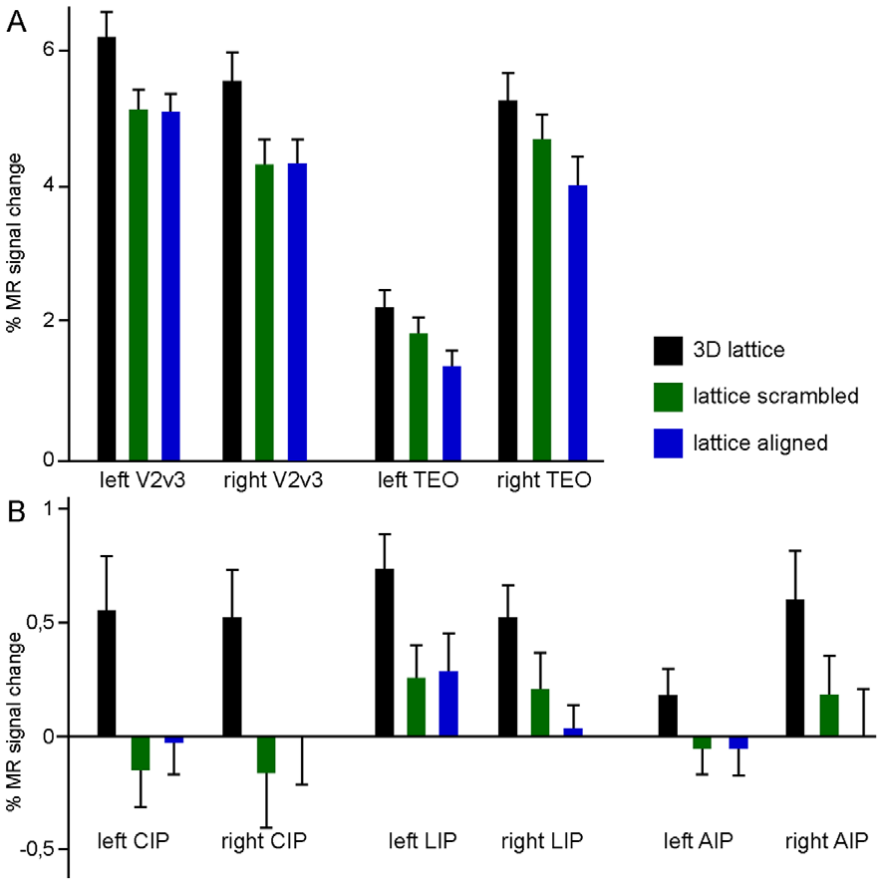
Activity profiles of regions involved in 3D SfT. Percent signal change from fixation baselines is plotted as a function of condition for left and right V2/V3, (ventral) TEO, CIP, anterior LIP, and AIP. Note the decrease in specificity of the profile as one moves more anterior in the IPS (compare CIP and AIP).

Figure 3 plots for illustrative purposes the activity profiles of left and right V2/V3, TEOv, CIP, LIP and AIP sites. While the overall level of activation is much higher in early visual areas and TEO, the parietal sites have a more specific profile. In particular CIP is only activated, relative to fixation, by the 3D shape condition and not by the 2D control conditions.

**Figure 4:**
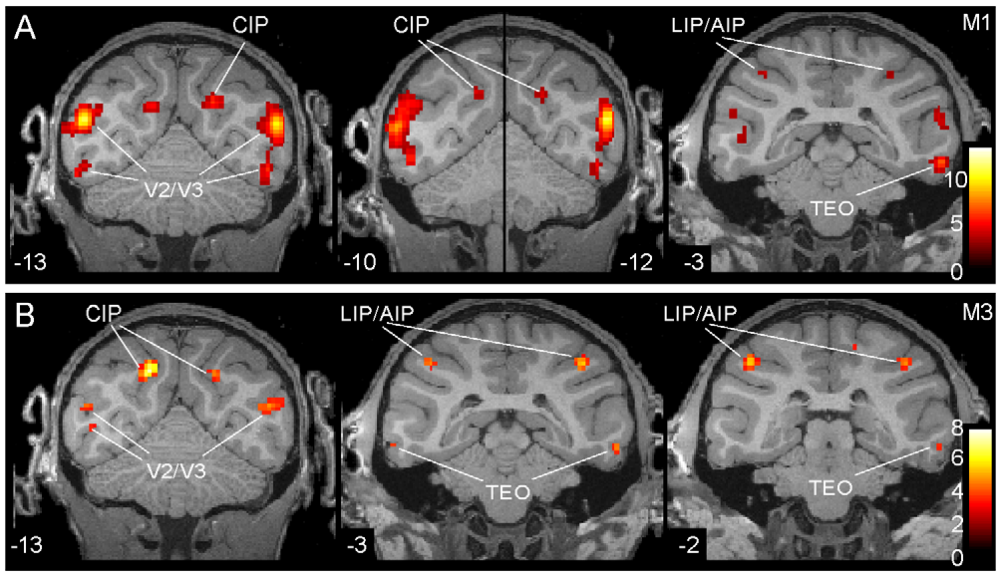
Statistical parametric maps of regions involved in 3D SfT. SPM t maps (single subject, p<0.001 uncorrected) from the conjunction of the two contrasts used in the 3D SfT experiment plotted onto coronal sections through the brains of M1 (A) and M3 (B). The middle panel in A is a composite of two coronal sections because the CIP activation reached maximum at slightly different anterior-posterior levels. Numbers indicate y coordinates, i.e. distances posterior from interaural plane.

These activation sites were observed not only in the group analysis but also in the single subject analysis, as shown in Figure 4. The V2/V3 activation is visible in the posterior coronal section (levels −13 to −10) in both animals. The posterior activation site in the lateral bank of IPS, corresponding to CIP is visible in both animals at the same levels. In the most posterior section of M1, the posterior activation site in the medial bank is visible. Finally the activation near the LIP/AIP boundary can be seen at levels −2 and −3 in both animals. At these levels also the activation in TEO around the PMTS can be observed.

### Cortical Regions Processing Depth Structure from Shading in the Monkey

To map the region involved in the extraction of depth structure from shading (3D SfS) we used the conjunction of four contrasts comparing 3D shaded condition to shaded blob, center shaded, pixel scrambled and uniform luminance conditions. This analysis (blue voxels in Figure 2; p<0.05, corrected) yielded a more restricted activation pattern than the 3D SfT test, in agreement with the results from the human study of [1]. Since the data of the two experiments were collected on different days, a direct comparison of the two activation patterns is not warranted. The number of voxels activated at p<0.05 corrected (Figure 2) equaled 31 in the SfS experiment compared to 476 in the SfT experiment. At p<0.001 uncorrected level (Figure S2) the numbers were 238 and 1285 respectively. Significant activation was observed only in V4 bilaterally and the left dorsal portion of TEO. The symmetrical TEO activation site in the right hemisphere was revealed by lowering the threshold to p<0.001. At this level activations were also observed in left AIP and CIP (Figure S2). The V4 activation was located near the tip of lunate and inferior occipital sulci, where central vision is represented [25], [26], [12], unlike what we observed in early areas for 3D SfT. The TEO activation for 3D SfS was located more dorsally than the TEO activation for 3D SfT, with hardly any overlap. It was located at the edge of the lower bank of the superior temporal sulcus (STS), in a position close to the recently identified retinotopic map of PITd (Map [1] in Fig. 7 of Kolster et al. [27]).

**Figure 5:**
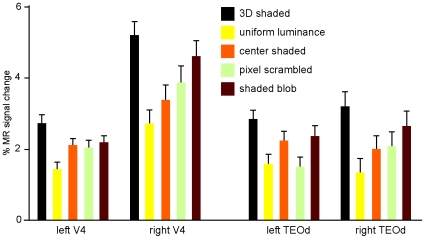
Activity profiles of regions involved in 3D SfS. Percent signal change from fixation baselines is plotted as a function of condition for left and right V4 and (dorsal) TEO.

The activity profiles of the V4 and TEO sites indicate (Figure 5) that the 2D control condition yielding the strongest activation was the shaded blob condition, as was the case in humans [1]. This is not surprising since TEO is known to be shape sensitive [28], [29] and V4 and TEO neurons are selective for shape elements [30], [31].

**Figure 6:**
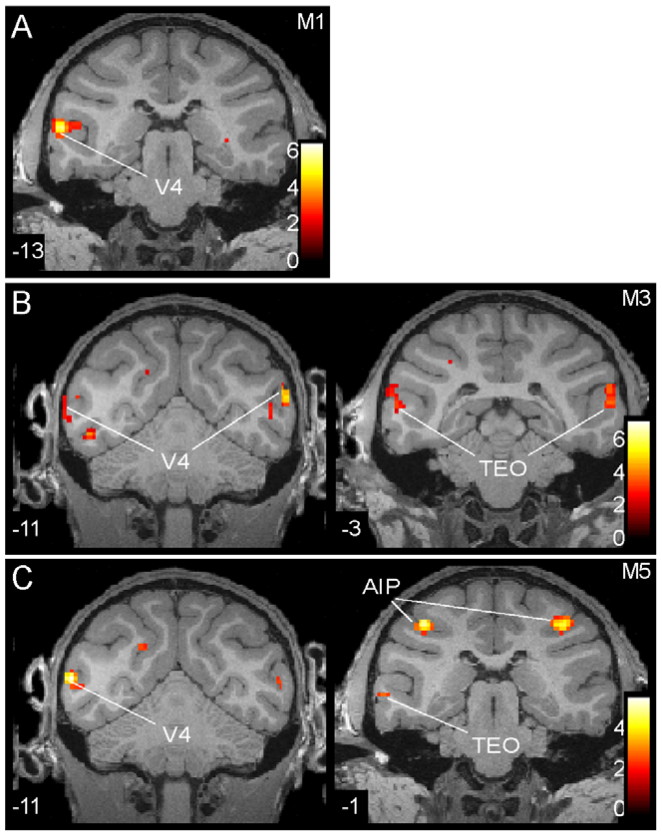
Statistical parametric maps of regions involved in 3D SfS. SPM t maps (single subject, p<0.001 uncorrected) from the conjunction of the four contrasts used in the 3D SfS experiment plotted onto coronal sections through the brains of M1 (A), M3 (B) and M5 (C).

Again, the activation pattern for 3D SfS was observed not only for the group, but also for the individual monkeys (Figure 6; p<0.001, uncorrected.). The V4 activation can be observed in all 3 monkeys at more posterior levels: −11 in M3 and M5 and −13 in M1. The activation in dorsal TEO at more anterior levels: −1 and −3 in monkeys M3 and M5. Notice that monkey M5 had a bilateral activation in AIP at p<0.001 uncorrected. It is noteworthy that this monkey had weak sensitivity for depth structure from disparity [22].

### Control Tests for the Difference between Texture and Shading

**Figure 7:**
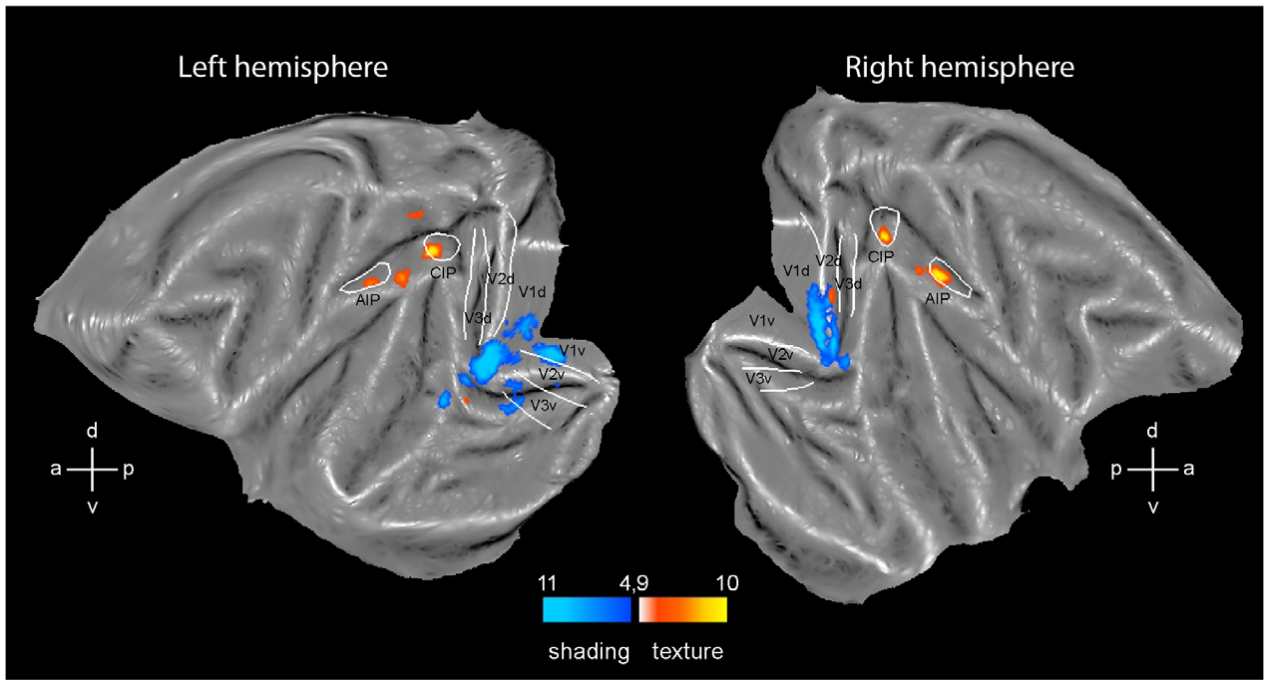
Control analyses. Flatmaps of the left and right hemisphere of monkey template (M12) brain (Caret software) showing regions significant (p<0.05 corrected) in the conjunction of two contrasts of the 3D SfS experiment (blue voxels, fixed effects, n = 3) and in four contrasts of the 3D SfT experiment (yellow to orange voxels, M3 single subject). Same conventions as in Figure 2.

The activation pattern for 3D SfT was clearly more extensive than that for 3D SfS (compare yellow and blue voxels in Figure 2). In particular there was little parietal activation by 3D SfS, exactly as was the case in the human study of Georgieva et al [1]. One possible confound, however, is that we used only a conjunction of 2 contrasts for the 3D SfT experiment and a conjunction of 4 contrasts for 3D SfS experiment. Thus the more restricted activation could be the consequence of a more stringent test for 3D SfS. Therefore, as a control, we restricted the conjunction for 3D SfS to 2 contrasts, those using the strongest 2D controls: shaded blob and the center shaded conditions. The result for the group of 3 monkeys is shown in Figure 7 (blue voxels; p<0.05, corrected). No parietal activation was observed for this restricted conjunction. The early visual activation increased, in particular in V1. This is to be expected as in humans the pixel scrambled condition is the control condition that removes most of the early activations (see Fig. 10 of Georgieva et al. [1]). As an additional control, we scanned all six texture conditions shown in Figure 1A in one monkey (M3). This allowed us to perform a conjunction of four contrasts, the same number as for the 3D SfS test. This analysis yielded a more restricted activation pattern compared to Figure 2, but nearly all activation sites left were located in the parietal cortex, including a bilateral activation of CIP, AIP and anterior LIP (Figure 7, yellow voxels). Thus the lack of 3D SfS sensitive activation in the IPS is not due to the analysis procedures we used.

### Processing of 3D and 2D Shape in Inferotemporal Cortex

**Figure 8:**
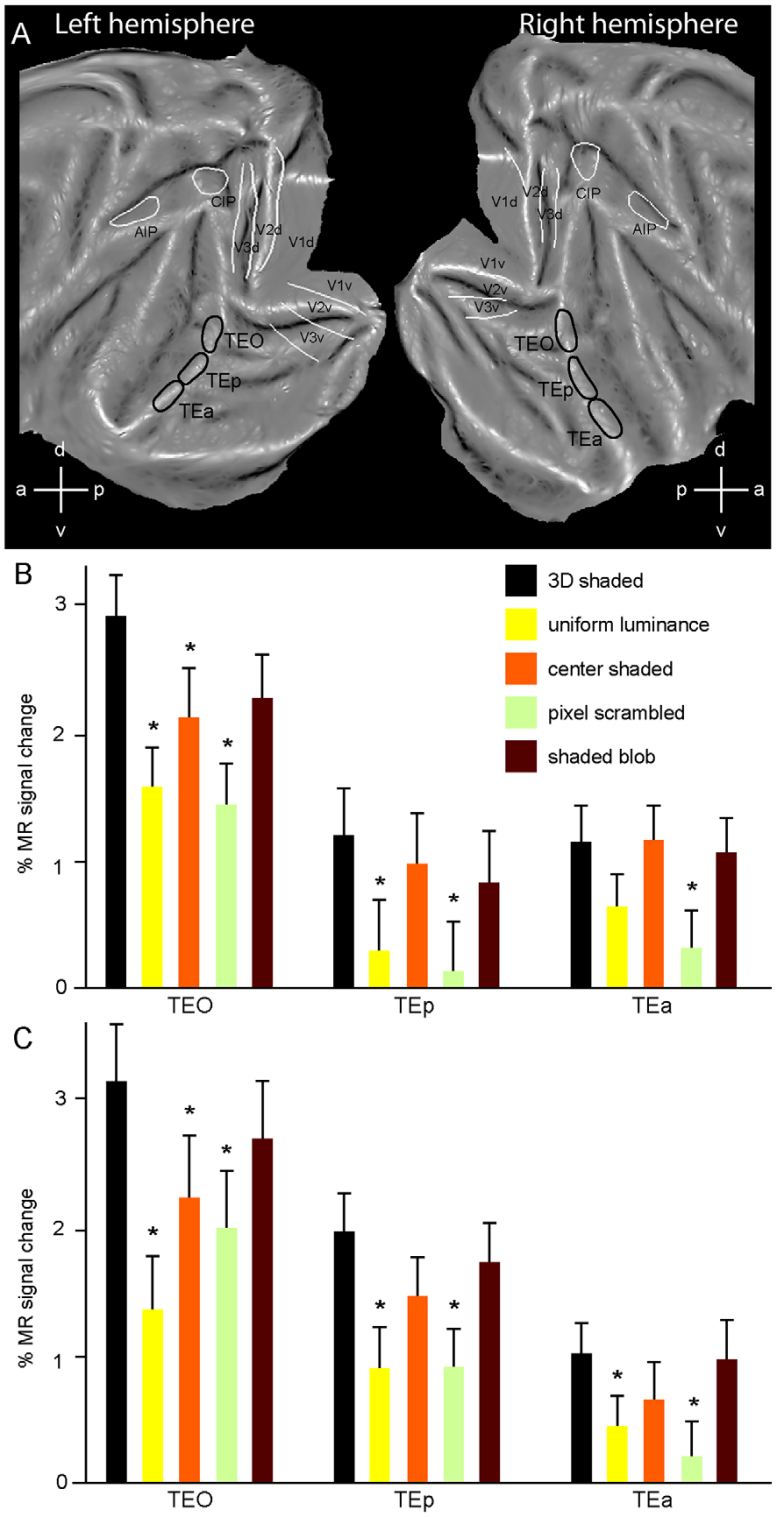
Activity profiles of subdivisions of inferotemporal cortex. A: definition of the 3 ROIs: TEO, posterior TE and anterior TE; B and C: activity profiles of the 3 ROIs of the left (B) and right (C) hemispheres (% MR signal change vs fixation baseline). Asterisks indicate conditions which differ significantly (p<0.05 one-way ANOVA followed by post hoc Bonferoni test) from 3D condition.

It is noteworthy that the infero-temporal regions involved in 3D SfT and 3D SfS were located in the posterior part of IT, just like in humans where 3D specific activations were observed in the posterior part of the LO complex [1]. To explore this effect further we defined 3 ROIs including TEO, posterior TE and anterior TE as indicated in Figure 8 (black encircled regions). These ROIs correspond to the parts of IT that have been explored by single recording using shading stimuli similar to those used in the present study. The activity profiles of these ROIs for the group of 3 monkeys are shown in Figure 8B (left hemisphere) and C (right hemisphere). The shaded blob condition, which was introduced as a control for selectivity for 2D shape, indeed activates these ROIs nearly as well as the 3D shaded condition. That the difference between these two conditions did not reach significance (one-way ANOVA, p<0.05, post-hoc Bonferroni correction) even in the TEO ROIs, indicates that the local maximum of the conjunction represented only a small part of TEO. This difference between the conditions systematically decreases as one moves forward in IT, exactly as it did in the human LO complex (see Fig. 11 of [1]).

## Discussion

Our results show that the regions specifically activated by 3D SfT belong to both the dorsal and ventral visual pathway, while mainly ventral regions are involved in extracting 3D SfS.

### Comparison with Earlier Monkey Studies

The most selective activity profile for 3D SfT is that of CIP. This cortical region was activated in the 3D condition and not in the control conditions of the 3D SfT experiment. This is in agreement with single cell studies which have reported neurons selective for the orientation of texture defined gradients in this cortical region [5]. Later studies from the same group suggest that neurons in the same region might also be selective for second order texture gradients. Selectivity for the orientation of first order texture gradients has also been reported for TEs neurons [7] and for MT/V5 neurons [8]. The TEs neurons selective for linear texture gradients were also selective for first order disparity gradients and these neurons occur in relatively small proportion in TEs. On the other hand TEs neurons selective for second order gradients of disparity which are much more frequent, hardly respond to second order texture gradients (Liu, Vogels and Orban unpublished). Thus a small proportion of TEs neurons will weakly be driven by the 3D texture stimuli of the present experiment yielding a population signal that is not strong enough to be detectable in the fMRI. A similar explanation may apply to MT/V5 in which few neurons are weakly driven by linear texture gradients [8]. It seems that in this case the signal was close to being detectable in the fMRI since a small activation was observed in the 3D SfT experiment in left MT/V5.

Our texture results are also in agreement with the study of Sereno et al. [9]. Using fMRI in the anaesthetized macaque these authors reported dorsal and ventral visual regions to be involved in 3D SfT. However, the exact identity of the areas involved seems to differ substantially from our results, at least in ventral cortex. In parietal cortex Sereno et al. [9] reported activation of LIP and to some degree in LOP which in all likelihood corresponds to CIP [22]. This fits relatively well with our observation although we also observed weak activation in AIP. In occipito-temporal cortex, however, Sereno et al. [9] report widespread activation, including that of MT/V5 and FST, but also four regions more anterior in IT or the STS. No activation was reported in TEO, which is the major site we observed in the present study. There are many differences between the two experiments, including in the state of the animals [32], in the type of stimuli, and in field strength, which might account for the differences in results.

As noted in the introduction little is known about the processing of shading to extract 3D shape in the monkey visual system. We observed activation in V4 and TEO, clearly posterior to the antero-posterior (AP) levels where Yamane et al. [4] reported 3D shape selective neurons (AP 6–22). In this latter study shading was systematically associated to disparity and frequently also texture, therefore it is possible that the proportion of neurons selective for 3D shape defined only by shading is too small to be detectable with the present fMRI technique.

### Comparison with Other Cues

The regions involved in processing 3D SfT bear clear resemblance with those involved in 3D structure from motion (SfM) and depth structure from disparity. Although the experiments were performed in different subjects, they indicate that all three cues engage both the dorsal and ventral stream [22]–[24], [33], [34, present study]. In parietal cortex, anterior LIP is involved in the processing of depth structure from all three cues, although the effects of the motion cue are very weak. The same holds true for AIP, although here the effects of both the motion and texture cues are weak too. CIP processes predominantly depth structure from texture and disparity. It has been suggested that for the latter cue the size of stimuli might be an important factor [22]. Unpublished fMRI experiments (Joly, Todd, Vanduffel and Orban unpublished) indicate that the MR response of CIP to linear texture gradients also increases with size. Finally there is indication that parts of the medial bank of IPS, perhaps corresponding to PIP, may also process depth structure from texture, disparity and even motion [22]. In the ventral stream 3D SfT engages only TEO, or at least its ventral part. Different occipito-temporal regions are engaged by depth structure from disparity (TEs) and from motion (MT/V5 and FST).

While texture as a cue for depth structure seems to be processed at least to some degree in ways similar to motion and disparity, shading seems to be processed in a very different manner. Only dorsal TEO and V4 were found to be involved and the parietal contribution to 3D SfS seems very restricted, although some IPS activation was present unilaterally at p<0.001 uncorrected. In general negative results in fMRI experiments are difficult to interpret, as there might be technical reasons for a lack of activation. This is not likely in the present experiment as we use a surface coil and the parietal cortex is immediately below the coil. In addition we use a contrast agent which increases the sensitivity by a factor of five compared to the BOLD effect used in humans [10]. Even if we accept the conclusion that a parietal activation is absent or very weak in the SfS experiment, this does not mean that parietal regions do not respond to images of 3D objects, but that the response to images of 3D objects is not larger than to images of 2D shapes. This also applies to area TE and is in keeping with the findings of Janssen et al. [35] who reported that neurons on the TE convexity responded equally to images of 2D and 3D objects defined by disparity. These findings seem consistent with the general view that object recognition involves predominantly 2D representations of objects [36].

It is noteworthy that when one considers all four cues available to extract depth structure, a clear difference between dorsal and ventral stream emerges, although again caution is required when comparing across experiments. In parietal cortex there is some evidence of partial overlap between the cues, at least at the areal level. In ventral cortex this is not true: each cue seems to be processed in separate regions(s): dorsal TEO for shading, ventral TEO for texture, MT/V5 and FST for motion and TEs for disparity. It is noteworthy that all regions except TEs lie in close proximity to each other and are located just posterior to the AP levels where Yamane et al. [4] reported neurons selective for 3D shape defined by multiple cues. It is therefore tempting to conclude that the different cues are extracted in separate regions in the vicinity of the posterior TE region, from which Yamane et al. [4] recorded, and then converge upon it.

### Comparison with Human Data

The present results are in excellent agreement with the results of the parallel study in humans [1]. This agreement is not surprising, since the same stimuli were used in the two experiments and the subjects were in a similar state: awake and passively observing the stimuli. There are, however, important differences between the two sets of experiments as we used a contrast agent in monkeys but no in humans, also the number of subjects, degree of smoothing and statistical analysis were different. Also in humans additional control experiments were performed in which subjects paid attention the 3D shape. Yet, in both species the 3D SfT stimuli engages both ventral and dorsal visual regions, while only ventral regions are involved in 3D SfS. Furthermore some of the regions involved in similar processes in the two species have already been reported to be homologous or at least functionally equivalent. Indeed there is mounting evidence for the homology between anterior LIP and human DIPSM and between posterior AIP and human DIPSA, reviewed in Durand et al. [37]. This equivalence is further supported by the present study. It has also been suggested that VIPS corresponds to CIP [37], [38], which is also supported by the present study. Finally this study and the earlier one [37] lend some support to the view that the fourth human IPS area involved in motion and shape processing, POIPS [39], might correspond to a medial bank area in the monkey, such as PIP. It is noteworthy that in both species the texture cue is clearly stronger in the posterior than in the anterior parietal regions (compare Fig. 8 of [1] with Figure 3 of the present study). A note of caution is warranted at this point. In the monkey AIP, CIP and LIP are considered single areas, although subdivisions of LIP have been proposed [40], [41], [22]. In the human we have defined functional regions and these probably correspond to multiple cortical areas, as defined eg. by retinotopy [42]. Hence the functional equivalence and homology between human and non human primates will require further refinement as the definition of cortical areas in both species progresses.

Also in occipito-temporal cortex there is a good agreement between the human study of Georgieva et al. [1] and the present study, since globally the human region of posterior ITG and its transition into IOG might correspond to TEO [43]. However, in humans partial overlap between the two caudal ITG regions involved in 3D SfT and 3D SfS respectively was observed, while in the present study the two sites were completely segregated within TEO. The partial overlap in the human study might be due to the averaging across subjects and smoothing, which induce a spurious overlap between functional activations.

In conclusion, in macaque monkey, as in humans, both parietal and occipito-temporal regions are involved in the extraction of depth structure from texture, while predominantly occipito-temporal regions are involved in extracting depth structure from shading. This opens up the path to single cell exploration of these visual processes.

## Supporting Information

Figure S1 All eleven different 3D objects used in the 3D shaded condition.
(5.85 MB TIF)

Figure S2 3D SfT and 3D SfS sensitive regions. Flatmaps of the left and right hemisphere of monkey template (M12) brain (Caret software) showing regions significant (fixed effects, p<0.001 uncorrected) in the conjunction of contrasts of the 3D SfT experiment (yellow to orange voxels, n = 2) and 3D SfS (blue voxels, n = 3) experiment. Same conventions as Figure 2.
(6.91 MB TIF)
