# Supplementary figures and images for: The Extraction of Depth Structure from Shading and Texture in the Macaque Brain
Source: PLoS One. 2009 Dec 14;4(12):e8306. doi: 10.1371/journal.pone.0008306 (PMC2789404; doi:10.1371/journal.pone.0008306)

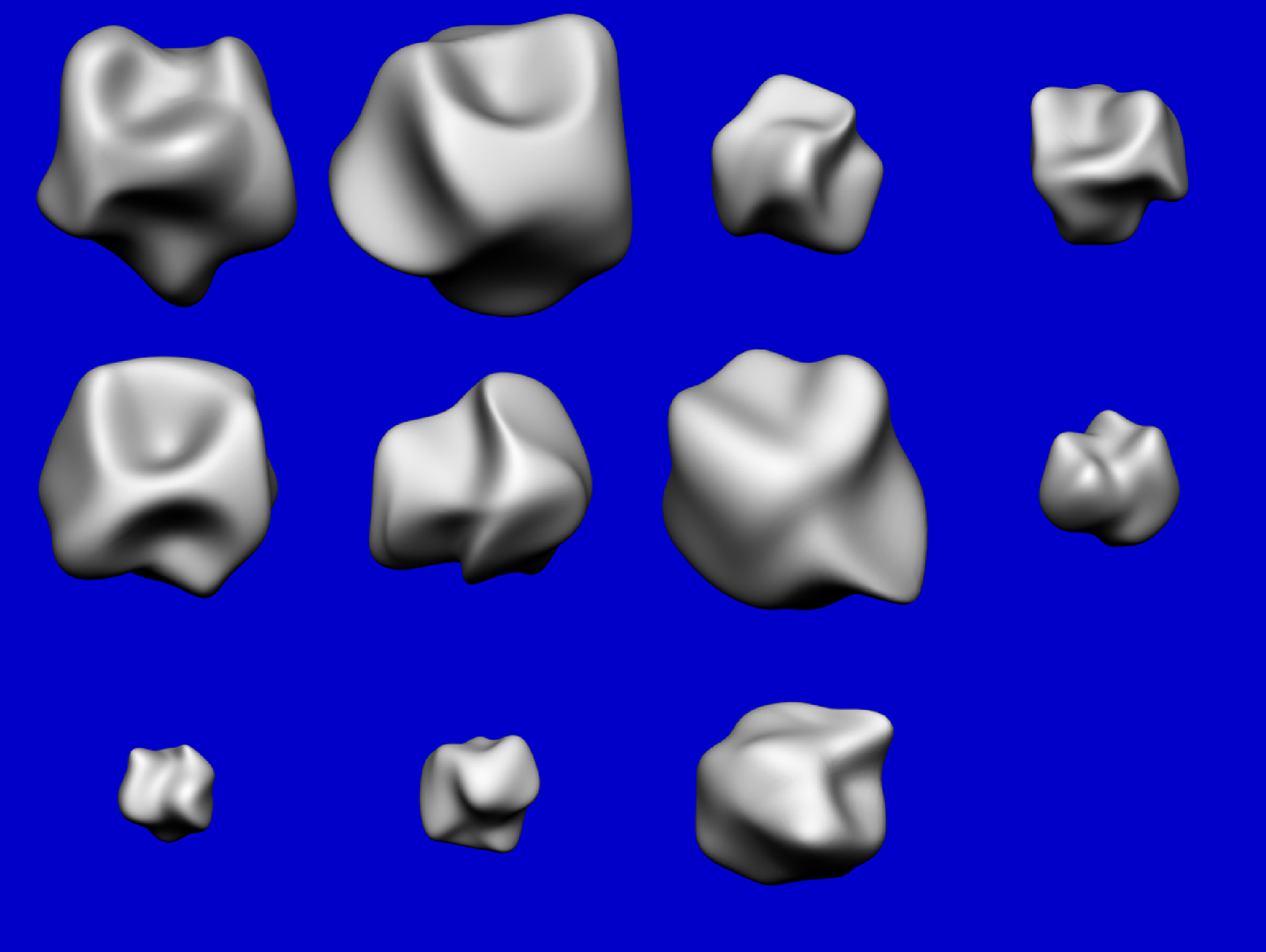

Supplement: Figure S1 — All eleven different 3D objects used in the 3D shaded condition. (5.85 MB TIF) [file pone.0008306.s001.tif]

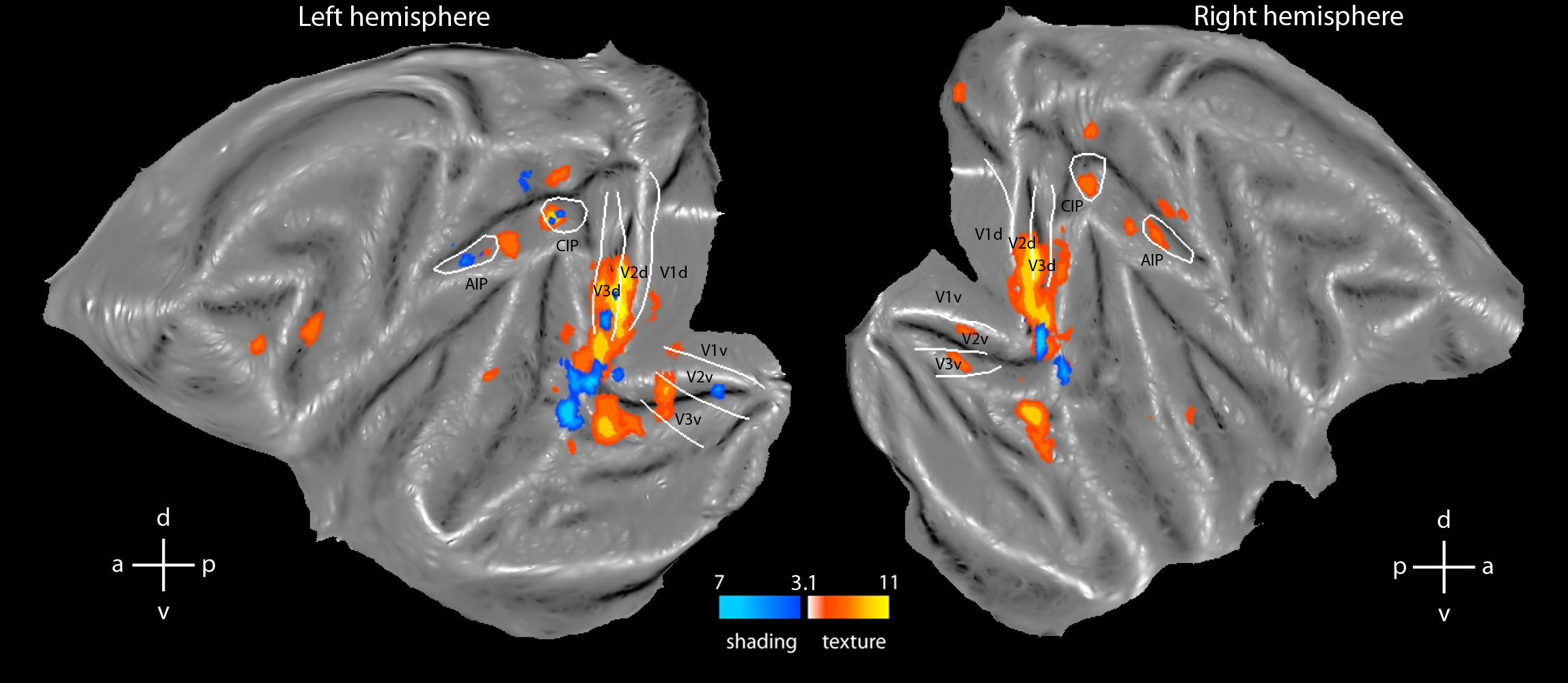

Supplement: Figure S2 — 3D SfT and 3D SfS sensitive regions. Flatmaps of the left and right hemisphere of monkey template (M12) brain (Caret software) showing regions significant (fixed effects, p<0.001 uncorrected) in the conjunction of contrasts of the 3D SfT experiment (yellow to orange voxels, n = 2) and 3D SfS (blue voxels, n = 3) experiment. Same conventions as Figure 2. (6.91 MB TIF) [file pone.0008306.s002.tif]
